# Paramedic management of back pain: a scoping review

**DOI:** 10.1186/s12873-022-00699-1

**Published:** 2022-08-09

**Authors:** Simon P. Vella, Qiuzhe Chen, Chris G. Maher, Paul M. Simpson, Michael S. Swain, Gustavo C. Machado

**Affiliations:** 1 Institute for Musculoskeletal Health, The University of Sydney and Sydney Local Health District, Sydney, NSW Australia; 2 Sydney Musculoskeletal Health, Faculty of Medicine and Health, The University of Sydney, Sydney, NSW Australia; 3 School of Health Sciences, Western Sydney University, Sydney, NSW Australia; 4 New South Wales Ambulance Service, New South Wales, Australia; 5 Department of Chiropractic, Faculty of Medicine, Health and Human Sciences, Macquarie University, Sydney, NSW Australia

**Keywords:** Back pain, Allied health personnel, Ambulances, Health service, Emergency medical services

## Abstract

**Background:**

Research examining paramedic care of back pain is limited.

**Objective:**

To describe ambulance service use and usual paramedic care for back pain, the effectiveness and safety of paramedic care of back pain, and the characteristics of people with back pain who seek care from paramedics.

**Methods:**

We included published peer-reviewed studies of people with back pain who received any type of paramedic care on-scene and/or during transport to hospital. We searched MEDLINE, EMBASE, CINAHL, Web of Science and SciELO from inception to July 2022. Two authors independently screened and selected the studies, performed data extraction, and assessed the methodological quality using the PEDro, AMSTAR 2 and Hawker tools. This review followed the JBI methodological guidance for scoping reviews and PRISMA extension for scoping reviews.

**Results:**

From 1987 articles we included 26 articles (25 unique studies) consisting of 22 observational studies, three randomised controlled trials and one review. Back pain is frequently in the top 3 reasons for calls to an ambulance service with more than two thirds of cases receiving ambulance dispatch. It takes ~ 8 min from time of call to an ambulance being dispatched and 16% of calls for back pain receive transport to hospital. Pharmacological management of back pain includes benzodiazepines, NSAIDs, opioids, nitrous oxide, and paracetamol. Non-pharmacological care is poorly reported and includes referral to alternate health service, counselling and behavioural interventions and self-care advice. Only three trials have evaluated effectiveness of paramedic treatments (TENS, active warming, and administration of opioids) and no studies provided safety or costing data.

**Conclusion:**

Paramedics are frequently responding to people with back pain. Use of pain medicines is common but varies according to the type of back pain and setting, while non-pharmacological care is poorly reported. There is a lack of research evaluating the effectiveness and safety of paramedic care for back pain.

**Supplementary Information:**

The online version contains supplementary material available at 10.1186/s12873-022-00699-1.

## Introduction

Back pain is the leading cause of years lived with disability worldwide and one of the most common reasons to call ambulance services [1, 2]. In Australia, one-third of patients with back pain arrive at the emergency department via ambulance [3]. The initial paramedic management of these patients may influence the subsequent care in the emergency department or inpatient units. Despite high rates of use, it is still unclear how ambulance services and paramedic clinicians are managing back pain cases [3].

Back pain is burdensome on the emergency healthcare system [4]. People with back pain who arrive at the emergency department by ambulance use more health services compared to those who arrive via their own means [3]. For example, back pain presentations that arrive by ambulance are more likely to receive lumbar imaging, opioid medications and hospital admission regardless of hospital setting (e.g. public or private hospital) [3, 5]. These back pain presentations via ambulance are an average of AUD$449 more costly to the hospital system when a patient is discharged, and an average of AUD$1,812 more costly when a patient is admitted to hospital, compared to non-ambulance presentations [6]. Interestingly, these presentations are less likely to be triaged as ‘emergency’ or ‘urgent’ patients compared to those who arrived using other modes [5].

Several guidelines exist to manage back pain in primary care [6]. Some of this guidance can be applied in the emergency department [7, 8], and potentially to paramedic care. However, it is currently unclear whether primary care guidelines apply to paramedic settings. To date, there has been no review summarising the evidence on paramedic management of back pain. Mapping this literature will aid understanding of their role in managing this condition, inform ambulance service policy and identify knowledge gaps in the field.

The objectives of this scoping review were to describe:

- The characteristics of patients with back pain who seek care from paramedics,
- The contribution of paramedic services to the total volume of health services a jurisdiction provides for back pain,
- Usual paramedic care for back pain, and
- The effectiveness and safety of paramedic care of back pain.

## Methods

We conducted a scoping review to assess evidence about paramedic services for back pain. The review followed the Joanna Briggs Institute (JBI) guide for scoping reviews [9] and adhered to the PRISMA extension for scoping reviews (PRISMA-ScR) [10]. The scoping review methods considered: research question/s, inclusion criteria (population, concept and context, study designs), search strategy, evidence of screening and selection, quality appraisal, data extraction and data analysis. The study protocol was registered through the Open Science Framework [11].

### Population

Eligible studies included people with back pain who received any type of paramedic care. There were no restrictions applied to age, duration, or type of back pain. Studies with mixed populations that provided data on back pain cases separately to other conditions were included in this review.

### Concept

We included primary studies that investigated usual paramedic care for back pain (e.g. pharmacological and non-pharmacological care), ambulance service use (e.g. the characteristics of patients with back pain who seek care from paramedics, the volume of back pain related calls that ambulance services receive, the frequency of back pain cases that receive vehicle dispatch and those that are transported to emergency departments) and effectiveness and safety of paramedic care.

### Context

Paramedic interventions provided on-scene (e.g. the person’s home) and/or during-transport to hospital.

### Eligibility criteria

Published peer-reviewed studies of any study design were eligible for inclusion [12]. To maintain a focus on paramedic management of back pain, any study that evaluated back pain experienced by paramedics was excluded. Conference abstracts and grey literature (such as government reports, policy statements, and unpublished research) were excluded.

### Data sources & searching

We searched MEDLINE, EMBASE, CINAHL, Web of Science, and SciELO from inception to July 2022. We used a validated paramedicine search filter [13] and the Cochrane recommended search terms for ‘back pain’ to design the search strategy (Appendix 1). No language or timeframe restrictions were applied to the search strategy.

JBI methodology recommends a 3-step approach to literature searching in scoping reviews. The first step, in which an initial search of MEDLINE would be completed to explore and identify suitable keywords and medical subject headings (MeSH) to develop the final search strings, was not undertaken due to the availability of the aforementioned validated filters. The final two steps (electronic searching and hand searching) were conducted as recommended.

All records identified through electronic database searches were exported to EndNote X9 (Clarivate, Philadelphia, US) and duplicates were removed. Article screening was conducted using Covidence (Veritas Health Innovation, Melbourne, Australia). Two reviewers (SV and QC) independently screened titles and abstracts for eligibility and then full text, with disagreements resolved by consensus or a third reviewer (GM). One author (SV) performed backward citation tracking of included studies to identify additional eligible studies. Articles reported in a language other than English were translated to English for review.

### Data extraction

One reviewer (SV) extracted all data into an Excel (Microsoft Corporation, US) spreadsheet and one of two reviewers (QC, GM) independently verified the data. Disagreements on extracted data were resolved by arbitration of a third reviewer (GM). When necessary, authors from individual studies were contacted by email to clarify data, or to provide separate back pain data from mixed populations. Extracted data were categorised into one of three groups:

i. Ambulance service use: the volume of ambulance service calls for back pain, the proportion of those that received ambulance vehicle dispatch, ambulance service response time, the proportion of cases transported to emergency department and those that were potentially avoidable, transport duration, and costs associated with paramedic care.
ii. Usual paramedic care: the proportion of people with back pain who received different types of paramedic care (e.g. opioid medicines, superficial heat therapy).
iii. Effectiveness and safety of treatment: results of randomised controlled trials evaluating paramedic interventions for back pain.

### Risk of bias of included studies

Methodological quality was appraised using the AMSTAR2 tool [14] for systematic reviews, PEDro scale [15] for randomised controlled trials, and the Hawker tool [16] for observational studies. The AMSTAR2 tool is a 16-item checklist that provides an overall confidence rating of high, moderate, low and critically-low based on weaknesses in critical domains (Appendix 2) [17]. Reviewers provide ‘yes’, ‘no’, and ‘partial yes’ (reported when part of, but not all of the AMSTAR2 criteria were met) responses regarding the manuscript meeting the AMSTAR2 criteria. The PEDro scale is a valid and reliable 11-item checklist [18, 19], total scores of 0–3 are considered ‘poor’, 4–5 ‘fair’, 6–8 ‘good’, and 9–10 ‘excellent’ [15]. The Hawker tool consists of 9-items and provides a total methodological rigor score ranging from 9 to 36 [16]. Total scores of 9–23 are considered ‘low’, 24–29 ‘medium’ and 30–36 ‘high’ quality [20].

### Data presentation & synthesis

Continuous data were summarised with means and standard deviations (SD) or medians and interquartile range (IQR), dichotomous data were summarised as counts/proportions. Randomised trials were summarised with the treatment effect size presented on a forest plot (without pooling) using Review Manager 5.4 (The Cochrane Collaboration, 2020).

## Results

### Description of included studies

**Fig. 1:**
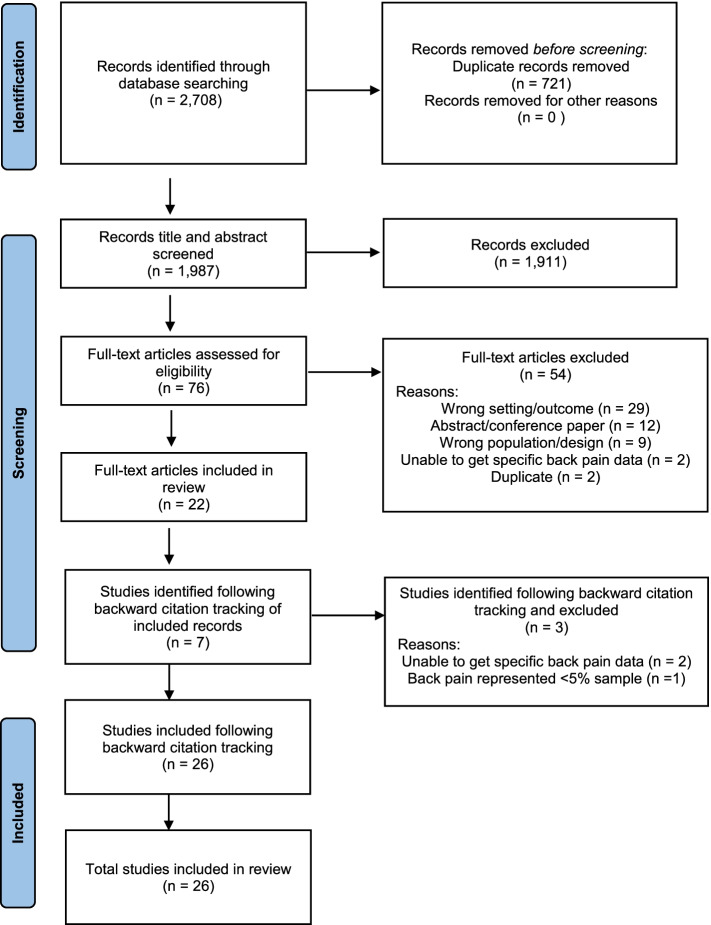
PRISMA diagram of study flow

The database searches retrieved 2708 records. After removal of 721 duplicates, titles and abstracts of 1987 records were screened for eligibility, and 1911 records were excluded. Full text screening of 76 records resulted in the inclusion of 22 articles. Backward citation tracking was performed on included studies and seven potentially eligible studies were identified. Upon full-text screening, four of these articles were included resulting in a total of 26 articles. The search flow is shown with reasons for exclusion in Fig. 1. PRISMA diagram of study flow.

### Study characteristics

Of the 26 included articles, there were three randomised controlled trials [21–23], 22 observational studies [2, 24–44] and one systematic review [45]. One cross sectional study reported the same data in two publications [42, 43], and the systematic review reported the results of one included trial, hence the reported data in this scoping review comes from the primary study [23]. The country of origin for the articles were United States (10) [26, 27, 30–33, 37–40], Australia (7) [2, 22, 24, 25, 29, 35, 45], United Kingdom (2) [28, 44], Austria (2) [21, 23] and Sweden (2) [34, 41], Spain (2) [42, 43] and Canada (1) [36]. Study characteristics are presented in Table 1.

**Table 1:** Study characteristics

Author	Design	Study setting-country	Type of paramedic service	Outcomes measured
Capsey *et al* [44]	Observational retrospective	Ambulance services—England	Ambulance service calls presenting with lower back pain	Service use Usual care
Alonso* et al* [42, 43]	Cross-sectional	Prehospital emergency setting-Spain	Home emergency nurse attending patients with lumbosciatica	Usual care Service use
Bertanlaffy *et al* [23]	RCT	Prehospital emergency setting—Austria	Paramedic managing first episode of acute LBP	Effectiveness of care Service use
Nuhr *et a*l [21]	RCT	Prehospital emergency setting—Austria	Paramedic managing first episode of acute LBP	Effectiveness of care Service use
Rickard *et al* [22]	RCT	Prehospital emergency setting- Australia	On-scene paramedic & ICP	Effectiveness of care Safety
Champagne-Langabeer *et al* [37]	Observational retrospective	Prehospital emergency setting—USA	EMS telehealth	Service use
Donen *et al* [36]	Observational prospective	Prehospital emergency setting—Canada	On-scene EMT	Usual care Safety
Infinger *et al* [26]	Observational retrospective	Prehospital emergency setting & transport to hospital—USA	Paramedic and EMT attending falls-related back pain patients	Usual care
Gill *et al* [28]	Observational retrospective	Emergency department trauma centre—Australia	Ambulance personnel managing patients with thoracolumbar fracture	Usual care
Eastwood *et al* [2]	Observational retrospective	Ambulance call service—Australia	Patients with ‘back symptoms’ who called ambulance service and received secondary telephone triage	Service use Usual care
Shah *et al* [30]	Observational retrospective	Ambulance call service—USA	Non-traumatic or non-recent back pain complaints made to 911 EMS call centre	Service use
Shah *et al* [40]	Observational prospective	Ambulance call service—USA	Non-traumatic or non-recent back pain complaints made to 911 EMS call centre	Service use
Eastwood *et al* [29]	Observational retrospective	Ambulance call service—Australia	Back pain case who received ambulance secondary telephone triage and were transported to ED by ALS & ICP	Service use
Eastwood *et al* [24]	Observational retrospective	Ambulance call service—Australia	Back pain cases who received ambulance secondary telephone triage by nurse or paramedic	Service use Usual care
Scott *et al* [39]	Observational retrospective	Ambulance call service—USA	Calls for non-traumatic back pain to EMS	Service use
Michael *et al* [32]	Observational retrospective	Ambulance call service—USA	Calls for non-traumatic and non-recent back pain to EMS	Service use Usual care
Eastwood *et al* [35]	Observational retrospective	Ambulance call service—USA	Back pain cases who received ambulance secondary telephone triage by nurse or paramedic	Service use
Eastwood *et al* [25]	Observational retrospective	Ambulance call service—Australia	Back pain calls to ambulance secondary telephone triage and receive emergency ambulance dispatch	Service use Usual care
Krumperman *et al* [38]	Observational retrospective	Ambulance call service—USA	Back pain calls from two EMS centres	Service use
Sporer *et al* [31]	Observational retrospective	Ambulance call service -USA	Prediction of prehospital intervention for back pain	Service use
Sporer *et al* [33]	Observational retrospective	Ambulance call service—USA	Back pain calls to EMS that received emergency dispatch and transport	Service use Usual care
Sporer *et al* [27]	Observational retrospective	Ambulance call service & transported by ambulance—USA	Back pain calls to EMS that were transported by ambulance	Service use Usual care
Hjalte *et al* [41]	Observational prospective	Ambulance services—Sweden	Back pain patients requesting ambulance transport	Service use
Hjalte *et al* [34]	Observational prospective	Ambulance services—Sweden	Back pain patients requesting ambulance service	Service use
Simpson *et al* [44]*	Systematic review	Prehospital	Paramedics managing acute pain	Usual care

Key: *RCT* Randomised controlled trial, *LBP* Low back pain, *ICP* Intensive care paramedic, *EMS* Emergency medical service, *EMT* Emergency medical technician, *ED*; Emergency department, *ALS* Advanced life support paramedic

^*^One study in this review was eligible to this scoping review and details regarding study characteristics are reported above under *Bertalanffy *et al.

### Quality appraisal of included studies

Quality appraisals of the included studies are presented in Table 2. Of the observational studies, 16 (73%) were graded as ‘high’ quality [2, 24–26, 28–33, 35, 37–40, 44], five (23%) as ‘medium’ quality [27, 34, 41–43], and one (4%) was ranked ‘low’ quality [36] according to the Hawker tool. The domain ‘ethics and bias’ scored poorly across all observational studies due to being retrospective cohort designs. The three randomised trials [21–23] were graded ‘good’ (i.e. PEDro score > 6) with deductions in the domains blinding of assessors, therapists and/or subjects or intention-to-treat analyses. The AMSTAR2 tool was used to appraise the one included systematic review [45] and was graded ‘moderate’ due to weaknesses in review methods, reporting of funding sources and publication bias and/or its impact on the study results.

**Table 2:** Quality appraisal of included studies using the Hawker, PEDro and AMSTAR 2 tools

Hawker tool		Items																					
**Author**		**Abstract & title**		**Introduction & aims**		**Method & data**	**Sampling**			**Data analysis**			**Ethics & bias**			**Results**	**Transferability or generalisability**	**Implications & usefulness**				**Score**	**Grade**
Capsey *et al* [44]		4		4		3	4			4			3			4	4	4				**34**	**High**
Donen *et al* [36]		3		2		2	2			1			1			4	2	2				**19**	**Low**
Infinger *et al* [26]		4		4		4	4			4			1			4	4	4				**33**	**High**
Champagne-Langabeer *et al* [37]		4		4		4	4			4			2			4	4	3				**33**	**High**
Alonso *et al* [42, 43]		4		3		4	4			1			1			4	4	3				**28**	**Medium**
Gill *et al* [28]		4		3		4	4			3			2			4	4	4				**32**	**High**
Scott *et al* [39]		4		4		4	3			4			2			4	3	3				**31**	**High**
Michael *et al* [32]		4		3		4	4			2			2			4	4	3				**30**	**High**
Krumperman *et al* [38]		4		3		4	4			4			2			4	4	4				**33**	**High**
Shah *et al* [30]		4		4		4	4			4			2			3	4	4				**33**	**High**
Shah *et al* [40]		4		4		4	4			4			1			4	4	4				**33**	**High**
Sporer *et al* [27]		4		3		4	4			1			1			4	4	3				**28**	**Medium**
Sporer *et al* [31]		4		3		4	4			4			2			4	4	3				**32**	**High**
Sporer *et al* [33]		4		3		4	4			2			2			4	4	3				**30**	**High**
Hjalte *et al* [41]		3		3		3	3			3			2			4	3	3				**27**	**Medium**
Hjalte *et al* [34]		4		4		3	3			2			2			4	3	3				**28**	**Medium**
Eastwood *et al* [2]		4		4		4	4			4			1			4	4	4				**33**	**High**
Eastwood *et al* [24]		4		3		4	4			2			3			4	4	4				**32**	**High**
Eastwood *et al* [35]		4		3		4	4			3			3			4	4	3				**32**	**High**
Eastwood *et al* [25]		4		3		4	4			2			3			4	4	4				**32**	**High**
Eastwood *et al* [29]		4		3		4	4			4			1			4	4	3				**31**	**High**
**PEDro Tool**		**Items**																					
		**1.1**	**1.2**		**2**	**3**		**4**		**5**		**6**		**7**		**8**	**9**		**10**		**11**		**Overall**
Rickard *et al* [22]		1	1		1	1		1		1		0		0		0	1		1		1		**7**
Nuhr *et al* [21]		1	1		1	1		1		1		0		0		1	1		0		1		**7**
Bertanlaffy *et al* [23]		1	1		1	1		1		1		1		0		1	1		0		1		**8**
**AMSTAR 2 tool**	**Items**																						
	**1**	**2**	**3**	**4**	**5**	**6**	**7**		**8**		**9**		**10**		**11**	**12**	**13**	**14**		**15**		**16**	**Overall**
Simpson *et al* [45]	Y	N	Y	PY	Y	Y	Y		PY		Y		N		Y	Y	Y	Y		N		Y	**Moderate**

*Key*: *PY* Partial yes

### Profile of patients

People who sought ambulance services, including those with back pain, were more likely to be female (median 54.4%, IQR: 52.9–58.1%) with a median age of 54.7 years (IQR: 44.3–58.0). The type of back pain that presented to ambulance services included; non-traumatic and non-recent back pain (i.e. duration > 6 h) [27, 30–33, 39, 40], first episode of acute back pain [21, 23, 45], lumbar radiculopathy [43], falls-related back pain [26], and thoracolumbar fracture [28]. One study categorised ‘lower back pain’ patients into one of three sub-categories including; spinal pain (e.g. serious spinal pathologies, nerve root compression and non-specific back pain), problem arising elsewhere (e.g. pain arising from somewhere other than the lower back) and deferred diagnosis (e.g. patient required further opinion) [44]. There were 1587 (47.9%) patients recorded as ‘pain arising elsewhere’, 1151 (34.7%) as ‘spinal pain’, and 471 (14.2%) as ‘deferred diagnosis’ that included 102 (3.1%) cases that were not recorded [44]. There was no definition of type of back pain in 11 studies [2, 22, 24, 25, 29, 34–38, 41] and only two studies [21, 23] reported duration of back pain, both as acute.

### Ambulance service use

The median (IQR, min–max) percentage of total ambulance calls that were due to back pain was 6.1% (1.4–10.1%, 0.6–12.5%). The highest call volume for back pain occurred on Sunday and Monday, and the lowest was on a Friday [44]. Back pain related calls peaked between 9 and 11am and dropped-off after 8 pm [44]. Most studies focused on evaluating low acuity conditions, with back pain ranked amongst the top 10 low acuity conditions in all studies and in the top 3 for over half the studies. The median (IQR, min–max) percentage of back pain calls that led to ambulance dispatch was 78.3% (69.6–87.1%, 61.1–95.9%) and an ambulance transported the patient to hospital for 16.1% (7.3–28.2%, 0.2–69.3%) of back pain calls. One study reported 66.8% of back pain cases as ED suitable (i.e. were triaged as a category 1,2 or 3 according to the Australian Triage Scale, were admitted to hospital, or died in ED) and 51.2% of back pain patients were admitted to hospital [29]. Appendix 3 reports data on ambulance service for back pain.

Response and attendance times were infrequently reported. Two studies [2, 40] reported mean/median ambulance dispatch times (i.e. time between ambulance service receiving call and dispatching an ambulance) of 7.7 min and 8.9 min. One study [43] reported mean ambulance attendance time (i.e. the amount of time that the paramedic spent on scene with the patient prior to transport to hospital or initiating referral to other services) of 16.0 min (SD 5.95).

No studies evaluated costs-associated with paramedic management of back pain.

### Usual paramedic care of back pain

Table 3provides data on usual paramedic care for back pain. Nine studies reported administration of analgesic medications for back pain including diazepam (benzodiazepine), diclofenac (non-steroidal anti-inflammatory drug), metamizole (analgesic), nitrous oxide (anaesthetic), opioids (including morphine, fentanyl, tramadol and codeine), ibuprofen and paracetamol. North East Ambulance Services in England reported nitrous oxide (24.3%) as the most frequently used medication for lower back pain followed by morphine (13.0%), paracetamol (8.5%), ibuprofen (2.4%) and other analgesics including co-codamol, codeine, diclofenac and ketamine [44]. A total of 902 (27.2%) lower back patients in this study were treated in the home-setting and 112 (3.4%) were taken to other health services such as medical centre, hospital ward, trauma and injury unit, and walk-in-centre [44]. In Spain, home emergency nurses were more likely to administer diazepam (65% of patients) and diclofenac (54% of patients) to manage low back pain at the patients’ home [43]. Two thirds (66%) of patients with thoracolumbar fracture that presented to an Australian hospital trauma centre had received prehospital opioids [28] and 76% of back pain cases that received emergency ambulance paramedic care (i.e. medium and high-acuity complaints) by Ambulance Victoria, Australia received an analgesic medication [25]. Lower use of analgesic medications were reported in low-acuity back pain populations where the eligibility criteria defined patients as having received basic paramedic support – no medications [27, 32, 34]. Despite this criteria, a small subgroup received advanced paramedic support including morphine (12% of patients).

**Table 3:** Pharmacological and non-pharmacological care provided for back pain

		Pharmacological care			
Author	Country	Data collection time frame	Proportion of sample with back pain N (%)	Type of back pain	Proportion of patients with back pain receiving medications or non-pharmacological treatments
Capsey *et al* [44]	UK	Aug 2016 – Jul 2017	3315 (100%)	Lower back pain	- Nitrous oxide (24.3%) - Morphine (13.0%) - Paracetamol (8.5%) - Ibuprofen (2.4%) - Other analgesics (1.7%)
Alonso *et al* [42, 43]	Spain	Jan 2012 – Apr 2016	237 (10.5%)	Lumbar radiculopathy	- Diazepam (64.9%) - NSAIDs (53.6%) - Metamizole (33.3%) - Opioids (11.4%) - Paracetamol (5.5%)
Infinger *et al* [26]	USA	Mar 2011—May 2011	154 (13.7%)	Falls-related back pain	- Opioid – Fentanyl (2%)
Eastwood *et al* [25]	Australia	Sep 2009 – Jun 2012	2,309 (9.7%)	Back pain (no definition provided)	- Received analgesia (76.2%)
Gill *et al* [28]	UK	Jan 2006 – Dec 2008	536 (100%)	All patients diagnosed with thoracolumbar fracture	- Opioid—Morphine (66%)
Sporer *et al* [27]	USA	Jan 2004 – Dec 2006	539 (0.8%)	Non-traumatic and/or non-recent back pain and back pain – patient not alert	Received analgesia: - Non-traumatic back pain (12%) - Non-recent back pain (8%) - Back pain- not alert (7%)
Sporer *et al* [33]	USA	Jan – Dec 2009	235 (0.6%)	Non-traumatic and/or non-recent back pain	- Received medication (22.6%)
Michael *et al* [32]	USA	Jan 2004 – Jul 2004	98 (6.1%)	Non-traumatic and/or non-recent back pain	- Opioids – Morphine (12%)
Donen *et al* [36]	Canada	NR	28 (11.7%)	NR	- Nitrous oxide (100%)
		Non-pharmacological care			
Eastwood *et al* [24]	Australia	Sep 2009 – Jun 2012	5,639 (12.7%)	NR	- Referred to alternate health service provider (16.1%) - Given self-care advice (10.6%)
Alonso *et al* [42, 43]	Spain	Jan 2012 – Apr 2016	237 (10.5%)	Lumbar radiculopathy	- Telecontact (80.5%) - Counselling (64.1%) - Interventions to stabilise emotions (27%) - Behaviour modification (24.9%) - Weight management (20.7%)
Eastwood *et al* [2]	Australia	Sep 2009 – Jun 2012	12,643 (11.8%)	Back symptoms	- Referred to alternate health service providers (13.5%) - Received care plan (0.5%)

Key: *NSAIDs* Non-steroidal anti-inflammatory drugs

Non-pharmacological strategies for back pain included telecontact (i.e. telephone consultation with a general practitioner), counselling and behavioural interventions (e.g. educational resources [46]) to allow adequate adherence to prescribed treatment, weight management advice, referral to alternate health services, including out-of-hours home-visiting doctor and nurse services, and hospital outreach programme that send allied health staff into the community, care plans, and self-care advice.

### Effectiveness and safety of paramedic care for back pain

**Fig. 2:**
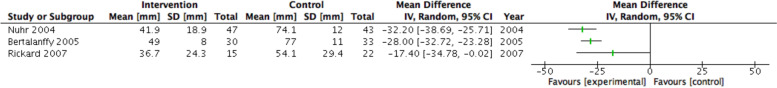
Effect of paramedic interventions on back pain Key: Rickard, 2007, intervention; intranasal fentanyl and control; Intravenous morphine

Three randomised controlled trials [21–23] reported on the effectiveness of analgesic treatment provided by paramedics during ambulance transport. One trial reported that active transcutaneous electrical nerve stimulation (TENS) was more effective than sham TENS in reducing acute back pain: treatment effect = -28.0 (95% CI -32.7 to -23.3) on a 100 mm visual analogue scale [23]. Another trial reported that active-warming was more effective than passive-warming to manage acute back pain: treatment effect = -32.2 (95% CI: -38.7 to -25.7) [21]. The third trial reported that intranasal fentanyl was more effective than intravenous morphine with a treatment effect of -17.4 (95% CI: -34.8 to -0.02) [22]. Effect sizes were taken ~ 30 min after administering the interventions. The treatment effect and sample sizes are presented in Fig. 2.

Two studies evaluated adverse reactions associated with the administration of medications nitrous oxide, fentanyl, and morphine in the prehospital setting for pain management [22, 36]. Neither of these studies reported safety of care specific to patients with back pain.

## Discussion

### Principal findings

This scoping review found that paramedics are frequently responding to and managing people with back pain. Back pain is in the top 10 reasons to call an ambulance service for low acuity conditions and in 78.3% of cases an ambulance is dispatched. The mean time from call to ambulance dispatch is ~ 8 min and 16% of calls for back pain receive transport to hospital, though transportation rates varied from 0.2% in low acuity settings to 69.3% in mainstream ambulance service. Approximately one third of back pain cases transported to the emergency department are potentially avoidable. Pharmacological management of back pain varies according to type of back pain and type of paramedic setting (e.g. home emergency nurse vs emergency ambulance paramedic). Non-pharmacological strategies are poorly defined and reported in the literature, and only three trials have evaluated effectiveness of paramedic treatments. No studies provided safety or costing data.

### Implications

We have summarised existing evidence investigating ambulance service use for back pain, usual paramedic care, and effectiveness of treatment. Studies on safety of care and costs-associated with back pain are lacking in this setting. Data on usual care and effectiveness of care can inform the development of specific back pain guidelines for paramedics, thereby reducing the use of inappropriate interventions. For example, current primary care guidelines for back pain recommend the use of NSAIDs (e.g. ibuprofen) and should be considered in ambulance guidelines, alongside non-pharmacological options such as hot and cold therapy [47, 48]. Ambulance service use data could inform ambulance service planning, training of staff and the use of alternate health pathways, such as referral to medical centres, general practitioners and allied health professionals. Additional industrial training or formal tertiary education of specialised paramedics in back pain management (e.g. paramedics specialising in primary care) could improve paramedic confidence and reduce risk mitigation in their decision-making processes compared to non-specialised paramedics who have operational pressures that limit their time on scene [49, 50]. Referral to alternate health pathways is often limited by clinic hours, strict criteria for referral, and accepting paramedics as ‘trusted’ referrers [51]. Additional training and referral to alternate health pathways could reduce overall costs on emergency healthcare services by reducing unnecessary ambulance dispatch and transport, hospital admission, and the cascade of events that follow (e.g. administration of opioids and lumbar imaging).

### Future research directions

There is limited research on patient profile, usual paramedic care and randomised trials evaluating treatment effectiveness and safety of care. The profile of patients with back pain using ambulance services needs to be identified. Most data come from patients visiting general practitioners and allied health professionals. In our review, the mean age of people using ambulance services for their back pain was 54 years and they were more likely to be female. The patient profile was only presented in 13 studies (and limited to age and gender) and not all the studies were representative of back pain cases. More data on back symptoms (e.g. level of pain, disability and duration of symptoms) and psychosocial aspects are needed. Investigating these areas will improve paramedic triage decision-making (i.e. identifying those who require and will benefit from paramedic care and ambulance service use).

There is a need to better understand how paramedics manage back pain. Currently, observational studies on usual paramedic care focus on administration of medications such as opioids, benzodiazepines, anaesthetics, NSAIDs and paracetamol to manage back pain. Most data on usual paramedic care comes from two papers [43, 44] and studies investigating large health systems (e.g. United States, Canada, Australia) are lacking. One study conducted in North East Ambulance Services in England reported the use of nitrous oxide in 24.3%, and morphine in 13% of patients with low back pain despite updated recommendations against the use of opioids (unless NSAIDs were ineffective or contraindicated) in primary care guidelines for low back pain [44, 47]. Additionally, according to the Spanish Society of Medicine of Family and Community, muscle relaxants are widely used in non-specific low back pain and may explain the high use of diazepam in the Alonso et al. paper. [43, 52] Benzodiazepines have been shown to provide no additional benefit to naproxen for acute low back pain [53] and clinical practice guidelines from primary care only recommend opioid-use when non-opioid analgesics have failed [47, 48, 54]. Despite these recommendations, ambulance service guidelines continue to focus on pharmacological intervention [55, 56]. Future research should evaluate health datasets from large health systems to investigate usual paramedic care of back pain.

There are only three trials investigating treatments delivered in a paramedic setting. While the three trials reported large treatment effects, lack of prospective registration, small samples, and concerns with risk of bias suggest that replication is required. Future trials need to investigate; i) commonly used drugs to manage back pain by ambulance services, and ii) outcomes and timepoints appropriate to the prehospital setting (e.g. within first hour). Furthermore, trials need to evaluate safety outcomes relating to paramedic care for back pain. This will assist in developing new and effective strategies to manage people with back pain in the prehospital setting.

Qualitative research exploring paramedic and patient perceptions of back pain and associated pain management strategies is needed to better understand paramedic management of back pain. This research should investigate; i) whether culture/ethnicity influences a patients’ perception towards back pain and the strategies used to manage their back pain, and ii) whether the perceptions between paramedics employed by non-transporting services and those of state-based emergency services influence management pathways such as patient transport, medication administration, and referral to alternate healthcare providers. Providing insight of the difference contexts of paramedic settings, the influence of these settings on management strategies, and how patient beliefs influence their management will help develop paramedic-specific pathways to manage back pain.

### Strengths and weaknesses of the study

This is the first scoping review, that we are aware of, that comprehensively maps the literature on paramedic management for back pain. It was performed following current guidance for scoping reviews [9]. We developed a sensitive search strategy that incorporated Cochrane recommended search terms for ‘back pain’ and a validated paramedicine search filter [13]. The scoping review design identified gaps in knowledge, for example, the need for more studies investigating usual paramedic care for back pain and trials testing the effectiveness and safety of paramedic treatments. Secondly, methodological quality of included studies was appraised using design-specific tools and provides insight into the quality of literature within the field.

There were two possible limitations of our review. Firstly, our search strategy did not include grey literature and as a result may have missed relevant government documents, policy statements, and conference abstracts. These documents were not identified in our search strategy despite being comprehensive and using sensitive search terms to minimise selection bias. Secondly, we included studies of mixed patient populations (e.g. back pain and other musculoskeletal pain) which limits the representativeness of data (e.g. patient profile) towards specific-back pain cases.

## Conclusion

Despite back pain being a common presentation to ambulance services and paramedic clinicians, there is a dearth of evidence to guide management in the prehospital setting. Future research is essential to identify effective strategies to manage people with back pain, to identify the characteristics of people who would benefit from ambulance services and evaluate the effectiveness and safety of paramedic care for back pain.

## Supplementary Information

**Additional file 1:** **Appendix 1**. Validated paramedicine search terms recommended by Olaussen et al^13^ and MEDLINE search result.

**Additional file 2**: **Appendix 2**. AMSTAR2 rating overall confidence in the results of a review.

**Additional file 3**: **Appendix 3**. Data on ambulance service for back pain.

## Data Availability

All data generated or analysed during this study are included in this published article [and its supplementary information files].

## Abbreviations

JBI: Joanna Briggs Institute
RCT: Randomized controlled trial
LBP: Low back pain
ICP: Intensive care paramedic
EMS: Emergency medical service
EMT: Emergency medical technician
ED: Emergency department
ALS: Advanced life support paramedic
IQR: Interquartile range
SD: Standard deviation
TENS: Transcutaneous electrical nerve stimulation
NSAIDs: Non-steroidal anti-inflammatory drugs
