# Supplementary material for: Paramedic management of back pain: a scoping review
Source: BMC Emerg Med. 2022 Aug 9;22:144. doi: 10.1186/s12873-022-00699-1 (PMC9361588; doi:10.1186/s12873-022-00699-1)
Supplement: Supplementary file 1 — Additional file 1: Appendix 1. Validated paramedicine search terms recommended by Olaussen et al13 and MEDLINE search result. [file 12873_2022_699_MOESM1_ESM.docx]

**Appendix 1. Validated paramedicine search terms recommended by Olaussen et al^13^ and MEDLINE search result.**

Paramedic Filter

Ambulances.sh OR Emergency Medical

Technicians.sh OR Air Ambulances.sh OR

emergency medical services.sh OR

paramedic*.tw OR ems.tw OR emt.tw OR

prehospital.tw OR pre-hospital.tw OR first

responder*.tw OR emergency medical

technicians.tw OR emergency services.tw

OR Ambulance*.tw OR HEMS.tw OR field

triage.tw OR out-of-hospital.tw

Ovid MEDLINE(R) <1946 to June Week 2 2021

1 Dorsalgia.mp. 99

2 exp Low Back Pain/ 23120

3 backache.mp. 3397

4 (lumbar adj pain).mp. 1350

5 coccyx.mp. 1409

6 coccydynia.mp. 115

7 sciatica.mp. 6410

8 exp Sciatic Neuropathy/ 7226

9 spondylosis.mp. 4531

10 lumbago.mp. 1300

11 back disorder*.mp. 577

12 radiculopathy.mp. 8296

13 exp back pain/ 40621

14 back pain.mp. 56307

15 low-acuity.mp. 426

16 non-serious.mp. 745

17 Ambulances.sh. 6291

18 Emergency Medical Technicians.sh. 5795

19 Air Ambulances.sh. 2922

20 emergency medical services.sh. 44331

21 paramedic*.tw. 7335

22 ems.tw. 11163

23 emt.tw. 18892

24 prehospital.tw. 11272

25 pre-hospital.tw. 3854

26 first responder*.tw. 1954

27 emergency medical technicians.tw. 735

28 emergency services.tw. 3419

29 Ambulance*.tw. 9539

30 HEMS.tw. 649

31 field triage.tw. 243

32 out-of-hospital.tw. 9700

33 1 or 2 or 3 or 4 or 5 or 6 or 7 or 8 or 9 or 10 or 11 or 12 or 13 or 14 or 15 or 16 77270

34 17 or 18 or 19 or 20 or 21 or 22 or 23 or 24 or 25 or 26 or 27 or 28 or 29 or 30 or 31 or 32 98729

35 33 and 34 293
