# Supplementary material for: Paramedic management of back pain: a scoping review
Source: BMC Emerg Med. 2022 Aug 9;22:144. doi: 10.1186/s12873-022-00699-1 (PMC9361588; doi:10.1186/s12873-022-00699-1)
Supplement: Supplementary file 2 — Additional file 2: Appendix 2. AMSTAR2 rating overall confidence in the results of a review. [file 12873_2022_699_MOESM2_ESM.docx]

**Appendix 2. AMSTAR2 rating overall confidence in the results of a review**

| **High**  *No or one non-critical weakness*: the systematic review provides an accurate and comprehensive summary of the results of the available studies that address the question of interest |
| --- |
| **Moderate**  *More than one non-critical weakness**: the systematic review has more than one weakness but no critical flaws. It may provide an accurate summary of the results of the available studies that were included in the revie |
| **Low**  *One critical flaw with or without non-critical weaknesses*: the review has a critical flaw and may not provide an accurate and comprehensive summary of the available studies that address the question of interest |
| **Critically low**  *More than one critical flaw with or without non-critical weaknesses*: the review has more than one critical flaw and should not be relied on to provide an accurate and comprehensive summary of the available studies |

*Multiple non-critical weaknesses may diminish confidence in the review and it may be appropriate to move the overall appraisal down from moderate to low confidence
