# Supplementary material for: Paramedic management of back pain: a scoping review
Source: BMC Emerg Med. 2022 Aug 9;22:144. doi: 10.1186/s12873-022-00699-1 (PMC9361588; doi:10.1186/s12873-022-00699-1)
Supplement: Supplementary file 3 — Additional file 3: Appendix 3. Data on ambulance service for back pain. [file 12873_2022_699_MOESM3_ESM.docx]

**Appendix 3. Data on ambulance service for back pain**

| **Study** | **Use of ambulance service** | **Time involved in paramedic service mean(SD)** | **RANK OF BACK PAIN AGAINST OTHER CONDITIONS** |
| --- | --- | --- | --- |
| Capsey *et al*^44^ | 3315/484,495 (0.68%) calls to ambulance service for lower back pain  2297/3315 (69.3%) received transport to hospital | NR | NR |
| Eastwood *et al*^25^ | 1,809/2309 (78.3%) received ambulance dispatch | NR | 2 |
| Alonso *et al* ^42, 43^ | 3/237 (1.3%) received emergency transport  1/237 (0.4%) received non-emergency transport | Ambulance attendance time: 15.99 (5.95) mins | 1 |
| Sporer *et al*^27^ | 539/69,541 (0.8%) calls to EMS for back pain | NR | NR |
| Eastwood *et al*^29^ | 1061/1,589 (66.8%) cases that received ambulance dispatch and emergency transport were ED suitable^#^  814/1589 (51.2%) cases were admitted to hospital | NR | 2 |
| Shah *et al*^30^ | 305/318 (95.9%) received ambulance dispatch | NR | NR |
| Michael *et al*^32^ | 98/1597 (6.1%) calls to EMS for back pain | NR | 5 |
| Sporer *et al*^33^ | 235/38,005 (0.6%) calls to EMS for back pain | NR | NR |
| Eastwood *et al*^2^ | 2,819 /12,643 (22.3%) received emergency transport  4,905/12,643 (38.8%) received non-emergency transport  7,724/12,643 (61.1%) received ambulance dispatch | Time from call to ambulance dispatch: 7.72 (5.00) mins | NR |
| Eastwood *et al*^35^ | 2,319/19,041 (12.2%) calls to ambulance service for back pain | NR | 2 |
| Krumperman *et al*^38^ | 5/590 (0.8%) calls to EMS for back pain | NR | NR |
| Scott *et al*^39^ | 1/601 (0.2%) received emergency transport | NR | 9 |
| Shah *et al*^40^ | 452/7,540 (6%) calls to EMS for back pain | Response time: 8min 53secs^ | NR |
| Eastwood *et al*^24^ | 1,821/5,639 (9.8%) received emergency transport  2,546/5,639 (24.6%) received non-emergency transport | NR | 1 |
| Champagne-Langabeer *et al*^37^ | 80/860 (9%) cases of non-traumatic back pain received ambulance transport | NR | 3 |
| Hjalte *et al*^34^ | 40/1,977 (2%) calls to ambulance relating to back pain | NR | 15 |
| Bertanlaffy *et al*^23^ | N/A | Transport time to hospital: TENS 24.8 (8.1) mins vs SHAM 26.2 (9.3) mins | NR |
| Nuhr *et a*l^21^ | N/A | Transport time to hospital: Active heating 24.8 (8.1) mins vs SHAM 26.2 (9.3) mins | NR |
| Rickard *et a*l^22^ | N/A | Transport time to hospital: INF 27.2 (15.5) min vs IVM 30.6 (19.1) min | NR |

Key: NR; not reported, mins; minutes, EMS; emergency medical services, ED; emergency department, N/A; not applicable, secs; seconds, TENS; transcutaneous electrical nerve stimulation, INF; intranasal fentanyl, IVM; intravenous morphine

^#^ a patient was considered ED suitable if they were triaged as a category 1,2 or 3 according to the Australian Triage Scale, were admitted to hospital, or died in ED.

^^^ response time represents all conditions

*Hjalte et al. report dataset in both of their studies^34,41^
